# Clinical Efficacy and Rehabilitation of Microscopic “Over the Top” for Bilateral Decompression in Degenerative Lumbar Stenosis: A Retrospective Study

**DOI:** 10.1155/2020/7174354

**Published:** 2020-12-09

**Authors:** Bin Lv, Sixin Sun, Haosheng Wang, Li Xiao, Tao Xu, Peng Ji, Jishan Yuan, Hua Ding, Jun Xie, Nan Meng, Lei Zhang, Minjie Hu, Qinyi Jiang, Lei Wang, Xiang Yao

**Affiliations:** ^1^Department of Orthopaedics, The Affiliated People's Hospital of Jiangsu University, Zhenjiang, Jiangsu Province 212002, China; ^2^Department of Orthopaedics, The Affiliated Taixing People's Hospital of Yangzhou University, Taixing, Jiangsu Province 225400, China; ^3^Department of Orthopaedics, The Second Hospital of Jilin University, Changchun, Jilin Province 130041, China; ^4^Department of Orthopaedics, The First Affiliated Hospital of Nanjing Medical University, Nanjing, Jiangsu Province 210029, China

## Abstract

**Background:**

Recently, “over the top” (also called ULBD; microscopic unilateral laminotomy for bilateral decompression) is a less invasive technique for symptomatic degenerative lumbar spinal stenosis (LSS), and this minimally invasive surgical technique has demonstrated favorable therapeutic outcomes. However, the risk of postoperative complications remains controversial.

**Objective:**

This study is aimed at determining the clinical efficacy and complication and rehabilitation of the microscopic “over the top” for degenerative LSS in geriatric patients. *Study Design*. This was a retrospective study. *Setting*. All data were obtained from the People's Hospital of a University.

**Methods:**

A retrospective analysis of 39 consecutive elderly patients treated for LSS by microscopic “over the top” between January 2016 and January 2018 was performed. A postoperative rehabilitation program for geriatric patients with restricted weight-bearing was instituted after the microscopic “over the top” treatment. Estimated blood loss, duration of operation, length of hospitalization, and total complications were also evaluated. The CT and MRI examinations of the lumbar spine were collected to evaluate the completeness of the nerve decompression. Clinical data were assessed at 6 months and 12 months after operation utilizing the visual analog scale (VAS), Oswestry Disability Index (ODI), and 36-Item Short-Form Health Survey (SF-36). Preoperative comorbidities, complications, and revision surgery were also recorded.

**Results:**

We enrolled a total of 39 degenerative LSS patients (27 male and 12 female patients, mean age of 75.8 ± 9.2 years). Twenty patients had one-level of degenerative LSS; thirteen patients had two-level of LSS; six patients had three-level of LSS. The average follow-up time in our study was 14.6 ± 7.8 months (range, 6-24 months). The overall complication rate was 10.2% (4/39), and the reoperation rates at one year were 2.5% (1/39). VAS back and leg pain score at 6 months were decreased to 1.8 ± 0.7 and 1.4 ± 0.6, respectively, and remained at 1.9 ± 0.3 and 1.2 ± 0.2 at 12 months, respectively. ODI scores improved significantly from 32.26 ± 6.82 to 11.44 ± 2.50 at 6 months and 10.56 ± 2.29 at 12 months. 36-Item Short-Form Health Survey scores revealed a significant improvement throughout follow-up. Postoperative complications included dural tear (*n* = 2), neurologic deficit (*n* = 1), and reoperation (*n* = 1). No infections or hematomas were reported. *Limitation*. Multicenter research is recommended to confirm our results and investigate the factors related to clinical and radiographic results.

**Conclusions:**

Microscopic “over the top” technique is a safe, effective option in the therapy of degenerative LSS and obtained satisfactory functional outcomes when coupled with aggressive rehabilitation, with a long recurrence-free recovery.

## 1. Introduction

The prevalence of degenerative LSS is expected to increase in the context of an increasing and aging population. Degenerative LSS is the most condition that causes clinical symptoms, such as lower back pain, leg pain, intermittent claudication, and neurological disorders. Symptomatic LSS can have a serious impact on functionality and quality of life and remains the most common indication for surgical intervention on the spine [1, 2]. The primary objective of surgical intervention for LSS is decompression of the symptomatic neural elements and to preserve or achieve mechanical stability [3].

Conventional laminectomy is a common surgical technique for providing adequate bony decompression and gets good-to-excellent outcomes in 64% of patients [4]. However, the destruction of the midline structures and the supra-/interspinous ligament caused by this treatment result in postoperative destabilization; it also leads to facet joint injury. Therefore, many geriatric patients are reluctant to undergo conventional laminectomy, based on the understanding that geriatric patients are at high risk for serious postoperative complications and poor outcomes.

**Figure 1:**
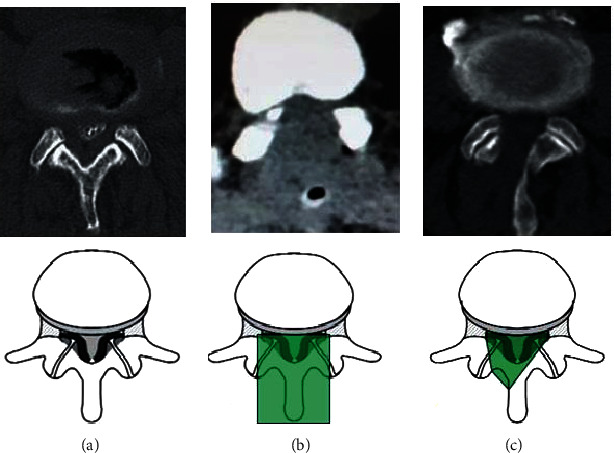
CT scans of traditional decompression and microscopic “over the top” procedures. (a) Spinal lumbar canal stenosis. (b) Traditional decompression approach removed most posterior elements and resected a large portion of the bilateral facet joints. (c) Microscopic “over the top” indicated that a laminotomy was performed by removing a portion of the superior and inferior laminae at the segment, and a small part of the medial facet. Deep cortical surface of contralateral lamina was undercut and drilling was extended to the contralateral medial facet.

Recently, “over the top” (also called ULBD; microscopic unilateral laminotomy for bilateral decompression) was initially described by Young et al. [5]. This surgical technique employs a unilateral exposure and muscle retraction, thus minimizing injury to the paraspinal muscles and the spinous process/interspinous ligament midline tension band structures [6] (Figure 1). This technique markedly reduces soft-tissue damage and intraoperative blood loss. Patients suffer from decreased postoperative pain and can usually be mobilized and discharged early.

Since geriatric patients are at a high risk for comorbidities, it is important to choose appropriate surgical interventions for this age group. For this reason, an appropriate selection of surgical technique should be used to answer two major problems: (1) Does this surgical technique decrease morbidity and mortality rates? (2) Does this surgical technique decompress the neural elements adequately to relieve symptoms? Therefore, this study is aimed at evaluating complication rates and clinical outcomes after a microscopic “over the top” approach in geriatric patients.

## 2. Methods

The study was approved by the Ethics Committee of the Jiangsu University and was performed in accordance with the Declaration of Helsinki. We retrospectively collected medical records of patients who underwent microscopic “over the top” lumbar decompression surgery between January 2016 and January 2018. All surgeries were performed using the “over the top” technique by one senior chief surgeon. Inclusion criteria included (01) 65 years of age or older; (2) patients with LSS confirmed by concordant imaging. Exclusion criteria were as follows: (1) a previous history of LSS surgery; (2) spinal instability, lumbar spondylolisthesis, and overweight patients with a body mass index (BMI) over 40; (3) psychiatric disorders such as schizophrenia, schizoaffective disorder, bipolar disorder and major depressive disorder, and peripheral neurological disease; (4) severe heart, lung, kidney, or liver disease. This study included 39 geriatric patients who received conservative treatment option before operation. Surgery was indicated if the conservative treatment had proved ineffective. The details of this study were explained to the patients, and all patients provided informed consent. Demographic and preoperative data (Table 1), including medical history, BMI, comorbidities, and American Society of Anesthesiologists (ASA) score, were documented. To simplify the presentation of medical history, we classified patients' comorbidities into several categories: cardiac, gastrointestinal, renal, pulmonary, and multiple comorbidity. Surgical data included the number of operated level, procedure time, and bleeding. Clinical outcomes included length of hospitalization, postsurgical complications, and revision surgery rate. The preoperative and postoperative cross-sectional area of the lumbar spinal canal was calculated from computed tomography (CT).

Flexion-extension lumbar radiographs were obtained again at 6 and 12 months postoperatively, and spinal instability was defined as a sagittal plane translation of ≥5 mm on flexion-extension radiographs (Radiological evaluation was judged by two observers). Postoperative follow-up consisted of the visual analog scale (VAS), Oswestry Disability Index (ODI) questionnaire, and 36-Item Short-Form Health Survey (SF-36) at 6 and 12 months. Patient-reported outcomes were collected via face-to-face assessment or telephone interviews.

### 2.1. “Over the Top” Technique

**Figure 2:**
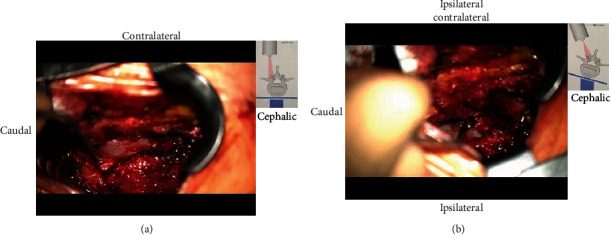
ULBD for spinal lumbar canal stenosis. (a) The inferior half of the L4 lamina has been drilled and the base of the spinous process to expose the ligamentum flavum bilaterally. (b) Decompression of the contralateral side. The tubular retractor is angled beneath the spinous process.

The microscopic decompression procedure has been previously described [7, 8]. In brief, we made a 3-5 cm midline incision after fluoroscopic confirmation of the surgical level. After skin incision, the multifidus muscle was dissected unilaterally from the spinous process and lamina using a Cobb elevator and retracted by a Taylor retractor. After detachment of paraspinal muscles, ipsilateral laminotomy was performed using a burr and Kerrison punches, followed by flavectomy using a microscope. To view the contralateral side, the operation table and microscope were tilted approximately 15 to 25°. For decompression, undercutting of the spinous process and contralateral lamina was performed using a burr and Kerrison punches, followed by flavectomy. After contralateral laminotomy and flavectomy, complete neural decompression was confirmed by the restoration of dural pulsation (Figure 2).

### 2.2. Rehabilitation

To obtain satisfactory lumbar functions, a systematic program of earlier active rehabilitation was carried out. The functional outcome analyses were performed by using a visual analog scale (VAS) for low back and leg pain, Oswestry Disability Index (ODI), and the modified MacNab criteria. Patient-reported outcomes were collected via face-to-face assessment or by using telephone interviews.

### 2.3. Statistics

All statistical analyses were carried out using SPSS software (Chicago, IL, USA). The Kolmogorov–Smirnov test was used to determine whether the distributions were significantly different. Data are shown as mean ± SD (standard deviation), the median (maximum–minimum) for ordinal variables, and the frequency with percent for categorical variables. Significant differences between groups were assessed using a one-way analysis of variance. The significant differences between median values were test by the Kruskal–Wallis test. Categorical comparisons were the chi-square test. *P* < 0.05 was considered significant.

## 3. Results

There were 27 (69.2%) men, and 12 (30.7%) women patients with a mean age of 75.83 ± 9.16 (range, 65-87) years were included in the study. Follow-up ranged from a minimum of 6 to 24 months (mean 14.6 ± 7.8 months). Twenty patients had one level of spinal stenosis; thirteen patients had two levels of stenosis; six patients had three levels of stenosis. Postoperative complications included dural tear (*n* = 2), neurologic deficit (*n* = 1), and reoperation (*n* = 1). No infections or hematomas were found in our study. Baseline characteristics of patients are in Table 1. Duration of operation, blood loss, length of hospitalization, and total complications were recorded in Table 2. There were four complications, including two dural tear (repaired primarily), one neurologic deficit (postoperative weakness of great toe dorsiflexion on the contralateral operative side, which was relieved spontaneously), and one symptom was no improvement (reoperation with laminectomy and fusion).

### 3.1. Radiographic Analysis

**Figure 3:**
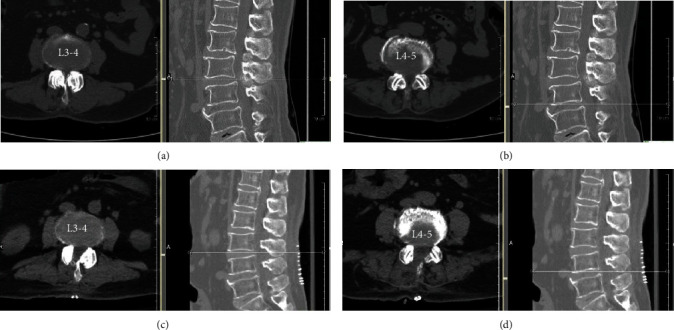
Preoperative computed tomography of L3-L4, L4-L5 stenosis (a, b), and postoperative computed tomography (c, d) obtained in one patient undergoing ULBD for 2-level stenosis.

**Figure 4:**
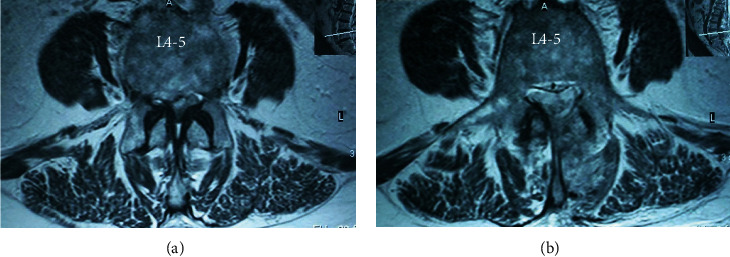
Preoperative (a) and postoperative (b) T2-weighted magnetic resonance imaging of L4-L5 stenosis.

CT scans demonstrated that the estimate of the cross-sectional area of the spinal canal was more significantly larger than preoperative data. Mean postoperative lateral recess height was higher than preoperative data (Figures 3 and 4); these results were statistically not significant at the follow-up period (Table 3). No abnormal movement in sagittal planes was observed on flexion-extension lumbar radiographs at 6 and 12 months (Table 3).

### 3.2. Clinical Outcomes

Low back and leg pain VAS scores demonstrated significant improvements in outcomes at the mean pre- and postoperative (3, 6, and 12 months) (Table 4). However, these improvements were not significant during the follow-up period. ODI scores decreased significantly from a preoperative score of 31.37 ± 8.61 to 12.44 ± 3.50 at 6 months and 12.30 ± 2.67 at 12 months, (Table 4), and we did not detect the significant differences at 6 months and 12 months.

SF-36 scores demonstrated a significant improvement at 6 months and 12 months after operation except for emotional role. There was no significant difference at both 6 and 12 months (Table 4).

## 4. Discussion

Low back pain (LBP) is the leading health issue of disability globally in the elderly population. Degenerative LSS is one of the most common causes of this condition. Raffo and Lauerman [7] showed that decompression contributed to the greater efficacy than nonsurgical treatments for symptomatic LSS patients in a randomized trial. However, age has been investigated as an independent risk factor for degenerative LSS, which was related to an increased risk of morbidity after open spine surgery [9]. Recent studies have shown that morbidity and complication rates were higher in geriatric patients compared with younger patients after surgery due to a variety of medical comorbidities [10, 11]. Therefore, it is important to choose appropriate surgical procedure, especially in geriatric patients, for whom the surgical challenges are their psychological and physiological factors. Thus, minimally invasive and efficient surgical intervention was adopted to decrease morbidity and mortality rates.

MIS approaches may produce less muscle-splitting to gain access to the spine and leave the midline structures intact, reducing intraoperative blood loss and relieving postoperative pain [12]. “Over the top” technique used the microendoscopic tubular-retractor system to preserve the facet joints and neural arch of the contralateral side, limiting postoperative destabilization and protecting the neural structures from extensive trauma. Ang et al. [13] retrospectively reviewed the clinical outcomes of 113 patients who underwent “over the top” and found that this technique was associated with reduced blood loss, shorter hospital stay, and lower complication rate. Although studies have confirmed similar results [14], the radiographic outcome and complications for elderly LSS patients have rarely been reported.

In the current study, the average age of patients was 75.83 years, and nearly half of our patients had two levels or multilevel LLS. We found that “over the top” technique was associated with significant improvement in VAS score, ODI score, and SF-36 at the postoperative follow-up period in most patients. Moreover, patients tolerated the surgical procedure well, even though some patients had medical comorbidities. Katz et al. [15] found that elderly patients with medical comorbidity and functional disability might not be very positive about treatment after decompression. Transfeldt et al. [16] reported that the procedural complication rate of open surgical decompression was 21%-40%.

In our study, the overall complication rate was 10.2%, and the results showed satisfactory outcomes. We speculated that the good results and few complications resulted from several inherent advantages of the MIS technique. Postsurgical stress response was found to lead to an imbalance in autonomic, endocrine, and immune systems. MIS technique can decrease inflammation and stress response after surgery. It was found to promote cardiovascular adverse events (hypertension, cardiac dysrhythmias, and myocardial infarctions) in the immediate perioperative period [17]. The frail elderly was suffering from multiple comorbidities, and limited physiologic reserve was vulnerable to stress. Therefore, the frail elderly may benefit from MIS procedures which reduce the risk of surgery.

Aging and medical comorbidities, such as cardiovascular diseases, can result in delayed wound healing that may jeopardize patient outcomes either by direct influence on spinal muscle rehabilitation [18]. “Over the top” results in smaller skin incisions that facilitate wound healing and decrease the risk of wound complications in the aging population. MIS procedure also can reduce blood loss [19]. The cardiovascular and pulmonary compensatory mechanisms are of limited capacity in older patients because age decreases the contractility and increases the stiffness of the left ventricle [20]. These alterations may impair the patient from tolerating large volume shifts, which can lead to life-threatening complications in geriatric patients.

The most common type of complication in “over the top” technique was dural tears. It is generally agreed that the opposite lateral spinal canal may require significant dural sac retraction through the unilateral approach, increasing the risk of dural tear or nerve injury. Here, dural tears occurred in 2 patients (4.4%) in our study. We used a 6-0 gauge with a tapered needle to repair dural defects in running locking stitch. The paraspinous muscles and overlying fascia were closed in two layers with nonabsorbable suture used in a watertight fashion. Two patients lied flat for 72 hours after operation and no infections and further headaches. The incidence of dural tears was similar to the previous study. Sidhu et al. [19] showed that the incidence of dural tears was 0%-18% for the microsurgical “over the top” technique. This rate was comparable to or lower than the incidence of dural tears reported in most series of decompression surgery for LSS [21]. To our knowledge, we cannot recommend this procedure in cases where severe radiculopathy symptoms are observed. Allowing contralateral microscopic visualization and using angled curets to create more space to perform the decompression surgery could reduce the incidence and severity of dural tears. When the dural injury happened, it must be meticulously sutured immediately to avoid CSF leakage.

For “over the top” technique, another main goal of surgery was adequate decompression of the neural elements. We utilized CT to measurements of the cross-sectional area of the spinal canal and lateral recess heights, and postoperative CT demonstrated decompression was enough. In contrast to our results, Thomé et al. [22] found that the ULBD was associated with less sufficient decompression than the bilateral laminotomy, even if the difference was not significant. This finding may suggest that the ULBD approach provides a worse view of the contralateral recess due to the limited exposure via a unilateral approach. However, Moisi et al. [23] concluded in their technical note that the ULBD approach could provide better visualization of the contralateral recess. Our selection of the decompression strategy using the ULBD technique was based on patients' symptoms and severity of LSS. For elderly patients with facet hyperplasia, ipsilateral facetectomy was routinely performed to obtain an adequate decompression for foraminal and lateral recess stenosis, it provided enough space and abduction angle to allow the undermining of the ventral aspect of the spinous process and contralateral lamina, and the posterior midline osteoligamentous structures and contralateral ligamentum flavum could be resected to expose the contralateral side, which allow contralateral facetectomy and provide complete decompression of the dural sac and contralateral nerve root.

During lumbar decompression surgery, the extent of preservation of the bilateral facet joint is an important factor for maintaining spinal stability [24]. Traditional standard decompression involves widely facetectomy and removal of the posterior spinal structures. As a result, the approach can lead to postoperative destabilization, which can lead to the need for spinal fusion and in turn is associated with increased comorbidities in geriatric patient. Mariconda et al. [25] showed that high rates of reoperation in open decompression ranged from 11% to 30%. In our study, we performed adequate decompression of the spinal stenosis, and no lumbar instability was found in the ULBD procedure. Miyazaki et al. [26].reported that the average percent facet joint preservation was significantly smaller than that in conventional decompression surgery. Further research has to be focused on the evolution of the stability of the treated spinal segment after different types of decompression. Microscopic “over the top” preserved 60%–83% of the facet joint on the approach side and >90% of the facet joint on the contralateral side. By contrast, the traditional approach retained ≤40% percent of the facet [27]. Therefore, we thought this MIS method can reduce the risk of postoperative spinal instability at the surgical site due to the satisfactory preservation of the facet joint.

## 5. Conclusions

Our experience supports the use of the ULBD as a less invasive technique for symptomatic LSS to conservative treatment that allows for adequate decompression of neural elements at the affected level. Even after symptom resolution, the general health and mental conditions of patients can also be significantly improved. The results of this study indicate that ULBD surgery is a safe and effective treatment for geriatric patients and does not increase the risk of complications.

### 5.1. Limitations

Our study has several limitations. First of all, we have our study with a limited number of patients, and the sample size was not sufficient to yield substantial effects. Second limitation, we were not able to compare beneficial results from ULBD in this age group with outcomes from those of an open surgical intervention in a similar age-grouped patient cohort. Finally, this was a retrospective study, and the indications for this surgical procedure were limited in patients with LSS. Additional studies with larger samples and longer follow-up periods should be performed to confirm the present results.

## Figures and Tables

**Table 1:** Baseline characteristics of the included patients.

Characteristics	(*n*, %, mean ± SD)
Age	75.8 ± 9.2
Gender (female/male)	12/27
BMI (kg/m^2^)	23.8 ± 2.3
Number of stenotic levels	
One level	20 (51.3%)
Two levels	13 (33.3%)
Three levels	6 (15.4%)
Laterality	
Unilateral	17 (43.6%)
Bilateral	22 (56.4%)
Level of stenosis	
L2 L3	4 (10.3%)
L3 L4	9 (23.1%)
L4 L5	16 (41%)
L5 S1	10 (25.6%)
Comorbidity	
Cardiac	10 (25.6%)
Endocrine	7 (17.9%)
Metabolic	5 (12.8%)
Gastrointestinal	4 (10.3%)
Pulmonary	6 (15.4%)
Renal	2 (5.1%)
Multiple comorbidity	7 (17.9%)
ASA score	2.72 ± 0.76
Follow-up (months)	14.6 ± 7.8

**Table 2:** The perioperative data and complications.

Parameter	Value (*n*, %, mean ± SD)
Duration of operation (min)	
1-level op	45.2 ± 9.4
2-level op	88.5 ± 17.4
3-level op	133.6 ± 37.5
Estimated blood loss (ml)	
1-level op	80.5 ± 12.8
2-level op	145.7 ± 55.7
3-level op	279.4 ± 70.3
Length of hospitalization (d)	5.4 ± 1.9
Total complications (*n*/rate)	4 (10.3%)
Dural tear	2 (5.1%)
Neurologic deficit	1 (2.6%)
Reoperation	1 (2.6%)

**Table 3:** Comparison of radiographic data preoperatively, at 6 months follow-up, and at 12 months follow-up for patients (mean ± SD).

	Preoperation	6 m	12 m	*P*-value
Cross-sectional area (mm^2^)	55.8 ± 26.6	138.8 ± 30.5	135.2 ± 35.7	P6 m − po = 0.001
				P6 m − 12 m = 0.27
Ipsilateral lateral recess height (mm)	1.8 ± 0.6	4.6 ± 2.2	4.4 ± 1.6	P6 m − po = 0.01
				P6 m − 12 m = 0.53
Contralateral lateral recess height (mm)	2.5 ± 0.7	4.1 ± 1.3	4.0 ± 0.9	P6 m − po = 0.01
				P6 m − 12 m = 0.59
Dynamic motion flexion-extension (mm)	2.2 ± 1.5	2.3 ± 1.7	2.5 ± 1.4	P6 m − po = 0.72
				P6 m − 12 m = 0.35

P6 m-po compared preoperation with 6 months follow-up; P6 m-12 m compared 6 months with 12 months follow-up.

**Table 4:** Oswestry Disability Index (ODI), visual analog scale (VAS), and 36-Item Short-Form Health Survey scores preoperatively, at 6 months and 12 months follow-up (mean ± SD).

	Preoperation	6 m	12 m	*P* value
ODI	31.37 ± 8.61	12.44 ± 3.50	12.30 ± 2.67	P6 m − po = 0.001
				P6 m − 12 m = 0.38
Back pain VAS	5.4 ± 1.6	1.8 ± 0.7	1.9 ± 0.3	P6 m − po = 0.001
				P6 m − 12 m = 0.26
Leg pain VAS	6.6 ± 2.8	1.4 ± 0.6	1.2 ± 0.2	P6 m − po = 0.001
				P6 m − 12 m = 0.17
SF-36				
Physical function	58.67 ± 5.23	72.45 ± 5.31	71.86 ± 4.68	P6 m − po = 0.001
				P6 m − 12 m = 0.51
Physical role	29.56 ± 8.77	49.01 ± 9.22	49.11 ± 8.75	P6 m − po = 0.001
				P6 m − 12 m = 0.69
Body pain	40.11 ± 4.11	68.25 ± 7.53	68.29 ± 7.75	P6 m − po = 0.001
				P6 m − 12 m = 0.44
General health	52.27 ± 4.25	58.26 ± 3.13	57.23 ± 4.54	P6 m − po = 0.01
				P6 m − 12 m = 0.39
Vitality/energy	41.73 ± 3.11	58.97 ± 5.21	59.12 ± 5.18	P6 m − po = 0.01
				P6 m − 12 m = 0.41
Social function	40.32 ± 5.41	51.54 ± 6.27	52.33 ± 6.09	P6 m − po = 0.01
				P6 m − 12 m = 0.36
Emotional role	63.62 ± 5.73	63.72 ± 5.73	63.69 ± 5.76	P6 m − po = 0.31
				P6 m − 12 m = 0.40
Mental health	43.36 ± 5.80	63.37 ± 5.77	63.34 ± 5.82	P6 m − po = 0.001
				P6 m − 12 m = 0.22

P6 m-po compared preoperation with 6 months follow-up; P6 m-12 m compared 6 months/with 12 months follow-up.

## Data Availability

Data will not be available because consent and ethical approval was not obtained for sharing. Additionally, all available data has been summarized in the text of the manuscript.

## Abbreviations

ULBD: Unilateral laminotomy for bilateral decompression
LSS: Lumbar spinal stenosis
VAS: Visual analog scale
ODI: Oswestry Disability Index
SF-36: 36-Item Short-Form Health Survey
ASA: American Society of Anesthesiologists.
